# Reducing onset-to-door times through the implementation of a prehospital large vessel occlusion bypass protocol: the Oahu experience

**DOI:** 10.3389/fstro.2026.1903852

**Published:** 2026-09-03

**Authors:** Chung-Huan Sun, Ian Chun, Kazuma Nakagawa, Stacy Brown, Samuel Tsappidi, Ferdinand Hui, Yi Zhang, Rony Salem, Guangxiang Zhang, Daniel Galanis, Matthew Koenig

**Affiliations:** 1 University of Hawai'i at Manoa John A Burns School of Medicine, Honolulu, HI, United States; 2 The Queen's Medical Center, Honolulu, HI, United States; 3 State of Hawaii Department of Health, Honolulu, HI, United States

**Keywords:** EMS triage, endovascular treatment, large vessel occlusion (LVO), stroke - diagnosis, treatment times

## Abstract

**Background:**

The Oahu large vessel occlusion (LVO) bypass protocol is a real-world island-wide EMS initiative designed to identify patients with suspected LVOs in the field and triage them directly to a Comprehensive Stroke Center (CSC) for endovascular therapy (EVT).

**Methods:**

A retrospective preimplementation-postimplementation study was conducted to investigate the impact of the bypass protocol on endovascular treatment times and patient outcomes between January 1, 2017 to January 1, 2023.

**Results:**

A total of 351 patients met inclusion criteria (102 patients pre-bypass vs. 249 patients post-bypass). In an interrupted time series analysis, there was a sustained 66-min reduction in onset-to-CSC door time (*p* = 0.0046), and a 41-min reduction in onset-to-reperfusion time (*p* = 0.08) in the post-bypass period with no changes in temporal trends. Interfacility transfers for EVT declined 5-fold (55.9% to 11.6%; *p* < 0.001) with no delay in onset-to-thrombolytic time (*p* = 0.13). In multivariate logistic regression modeling, longer onset-to-reperfusion times were independently associated with worse clinical outcomes, where every 30-min delay increased the odds of a higher 90-day mRS score by 6% (OR 1.062, 95% CI 1.001–1.125, *p* = 0.033). The post-bypass era, itself, however, was not associated with improved 90-day good outcomes (mRS 0–2: 40.2% vs. 44.6%; adjusted OR 0.98 [95% CI, 0.57–1.69]; *p* = 0.93) or a shift in 90-day mRS disability (adjusted OR 0.93 [0.60–1.41]; *p* = 0.72), though a nonsignificant trend toward improved 90-day excellent outcomes was observed (mRS 0–1: 23.5% vs. 34.5%; unadjusted OR 1.75 [1.01–2.90], *p* = 0.045; adjusted OR 1.51 [0.84–2.72], *p* = 0.17).

**Conclusions:**

Implementation of a system-wide EMS LVO bypass protocol successfully reduced onset-to-door and onset-to-reperfusion times within a uniquely closed catchment geography. The impact on clinical outcomes, however, remains to be seen.

## Introduction

Time to reperfusion is a well-established determinant of mortality, functional outcomes, and healthy-life years following endovascular therapy for ischemic stroke (Mazighi et al., 2013; Saver et al., 2016; Jahan et al., 2019; Almekhlafi et al., 2021). While substantial improvements have been made at reducing in-hospital “door-to-puncture” and procedure times, the prehospital component of “onset-to-door” time has remained stagnant (Sun et al., 2023). Systems of care initiatives have now shifted attention toward the prehospital phase of acute stroke management.

Emergency medical services (EMS) screening tools have been developed to identify patients with suspected large vessel occlusions (LVO) and facilitate direct transport to thrombectomy-capable centers, thereby reducing prehospital delays associated with interfacility transfers. Several retrospective studies have shown reduced disability and greater treatment eligibility among patients brought directly to Comprehensive Stroke Centers (CSC) (Jayaraman et al., 2020; Froehler et al., 2017; Sun et al., 2013; Kass-Hout et al., 2021). Two recent randomized controlled trials, RACECAT (Pérez et al., 2022) and TRIAGE-STROKE (Behrndtz et al., 2023) further evaluated the effectiveness of such EMS bypass strategies. Both studies demonstrated meaningful reductions in onset-to-puncture time, yet neither showed improvements in functional outcomes. Clinical equipoise remains as to whether prehospital bypass strategies truly confer clinical outcome benefit.

In this study, we evaluated the impact of an island-wide EMS LVO bypass protocol on both treatment times and patient outcomes over a 6-year period on Oahu, Hawaii - a uniquely controlled stroke system environment. The island has a single CSC staffed by the same cohort of neurologists and neurointerventionalists. All stroke patients are managed by a single EMS agency before transport to one of ten local hospitals or directly to the CSC. In contrast to prior studies where patients could be triaged to multiple thrombectomy-capable centers across open catchment areas, Oahu's closed geography and uniform provider network limits inter-system EMS and patient migration, providing a unique opportunity to assess the impact of a prehospital bypass strategy.

## Methods

### Oahu LVO bypass protocol

On August 1, 2019, the “Oahu LVO Bypass Protocol” was adopted island-wide for all EMS evaluations–voted into action by the Hawaii Stroke Coalition. In this protocol, patients with Cincinnati Stroke Triage Assessment (C-STAT) scores ≥2 were deemed “positive” for suspected LVO and bypassed the nearest local hospital enroute to the island's only CSC. Prior to this policy change, EMS protocols directed patients to be taken to the nearest hospital, regardless of stroke severity, unless designated as a level 1 trauma or at the discretion of the EMS provider.

### Setting

Oahu has a population of approximately one million people with a geographic catchment area of 597 square miles. The island is served by ten acute care hospitals, each with thrombolysis capabilities, but only one is designated as a thrombectomy-capable CSC. All decisions for mechanical thrombectomy are centrally triaged by a unified team of stroke neurologists and neurointerventionalists at the receiving institution, creating a coordinated regional system of care for LVOs (Supplementary Figure 1). During the study period, our institutional protocol for mechanical thrombectomy required baseline mRS of 0–2, NIHSS score ≥6, and an ASPECTS 6–10.

### Design and study population

Approval for the study was granted by our local institutional review board. A retrospective chart review was performed on all patients arriving by EMS who underwent endovascular treatment for LVO between January 1, 2017 and January 1, 2023. Patients were dichotomized into approximate 3-year pre-bypass (January 2017–August 2019) and three-year post-bypass cohorts (August 2019–January 2023) using August 1, 2019 as the protocol implementation date. Comparisons were made evaluating the impact of the bypass strategy on thrombectomy-related time metrics and clinical outcomes. Interisland transfers, non-EMS arrivals, posterior circulation strokes, inpatient strokes, and patients presenting more than 8 h from symptom onset were excluded (Supplementary Figure 2). The extended window population (8–24 h) was excluded under the assumption that delays to presentation (i.e., wake-up strokes or late symptom recognition) are not modifiable by EMS transport policies, and thus, outside the scope of the study.

### Outcome measures

Data was collected on baseline demographics, past medical history, stroke severity, radiographic findings including clot location and pre-treatment ASPECTS, thrombolytic therapy, angiographic results, symptomatic intracerebral hemorrhage, and clinical outcomes. Primary outcome measures were treatment times including: “onset-to-door” (defined as stroke onset to arrival at the CSC), “door-to-puncture” (defined as time of arrival at the CSC to arterial puncture), and overall “onset-to-reperfusion” (defined as onset time to successful TICI2b reperfusion). Secondary outcome measures included: 90-day good functional outcomes–defined as modified Rankin Scale scores (mRS) of 0–2; 90-day excellent outcomes (mRS 0–1); and the ordinal shift in 90-day mRS. mRS assessments were performed by our institution's certified stroke navigators who were blinded to the procedure results.

### Statistical analysis

A univariate analysis was performed comparing baseline characteristics between patients in the pre-implementation vs. post-implementation periods. All continuous variables were analyzed with Student *t*-tests based on the equality of variances. Time metrics were summarized using medians and interquartile ranges, and compared via Mann–Whitney *U*-tests. Categorical and ordinal variables were also compared using χ^2^ tests and Mann–Whitney *U*-tests, when appropriate.

An interrupted time series analysis (ITS) (Wagner et al., 2002) was utilized to evaluate the impact of the LVO bypass protocol on “onset-to-door,” “onset-to-reperfusion” and “onset-to-thrombolysis” times between the pre-implementation and post-implementation time periods. Treatment times were grouped by quarter averages. The ITS estimated the change in level for treatment times after implementation, as well as estimated any change in slope over time. The time series data was further assessed using Durbin-Watson statistic to assess for any features of autocorrelation that would confound changes in treatment times to temporal trends, rather than policy change.

The association between bypass era and good or excellent 90-day outcomes was initially evaluated using univariate analysis. A binary logistic regression model was then created to adjust for known predictors of outcome including age, baseline NIHSS, clot location, reperfusion status, and symptomatic hemorrhage. Given concerns for collinearity, “onset-to-reperfusion” times and bypass era were not included in the same models. A shift analysis was subsequently performed across the 7-point range of 90-day mRS scores using ordinal logistic regression modeling and adjusted for predictors of outcome. Odds ratios and 95% confidence intervals are reported. The goodness-of-fit of the models were confirmed using Hosmer-Lemeshow testing and proportional odds assumption, respectively. All statistical analyses were performed using IBM SPSS version 31 and SAS 9.04.

## Results

Between January 1, 2017 and January 1, 2023, 351 patients met inclusion criteria as confirmed anterior circulation LVOs treated within 8 h of symptom onset and transported to our CSC via local Oahu EMS. There were 102 patients in the pre-LVO bypass cohort vs. 249 patients in the post-LVO bypass cohort. Patients had a mean age of 70.3 ± 13.0 years old with a median NIHSS of 18 (IQR 13–23). Baseline demographics are summarized in Table 1. There were no significant differences in age, ethnicity, stroke severity (NIHSS), or post-endovascular symptomatic hemorrhages between the pre- and post-implementation cohorts. Notably, a higher proportion of patients in the post-bypass era had M2 occlusions (26.1% vs. 12.7%), achieved successful reperfusion (94.3% vs. 83.3%), and had good pre-treatment ASPECTS (88.3% vs. 77.5%).

**Table 1:** Demographic data of pre-LVO bypass vs. post-LVO bypass thrombectomy patients.

Characteristics	Pre-bypass era, *n* = 102	Post-bypass era, *n* = 249	*p*-value
Age, mean (STD)	69.5 (13.7)	71.7 (13.7)	0.175
Female, No. (%)	40 (39.2%)	123 (49.4%)	0.082
Atrial Fibrillation, No. (%)	46 (45.1%)	111 (44.6%)	0.929
Hypertension, No. (%)	82 (80.4%)	216 (86.7%)	0.131
Hyperlipidemia, No. (%)	57 (55.9%)	174 (69.9%)	0.012
Diabetes, No. (%)	29 (28.4%)	82 (32.9%)	0.410
Smoker, No. (%)	29 (28.4%)	71 (28.5%)	0.988
Ethnicity			0.613
Asian, No. (%)	49 (48.0%)	135 (54.2%)	
NHPI, No. (%)	24 (23.5%)	53 (21.3%)	
White, No. (%)	27 (26.4%)	52 (20.9%)	
Other, No. (%)	2 (2.0%)	9 (3.6%)	
NIHSS Score, mean (STD)	18.3 (6.9)	18.0 (6.9)	0.660
Clot Location, No. (%)			0.010
ICA-T	34 (33.3%)	56 (22.5%)	
M1	55 (53.9%)	128 (51.4%)	
M2	13 (12.7%)	65 (26.1%)	
Good ASPECTS 8-10, No. (%)	79 (77.5%)	220 (88.3%)	0.015
IV Thrombolytic Treatment, No. (%)	73 (71.6%)	157 (63.1%)	0.127
Successful Reperfusion TICI≥2b, No. (%)	85 (83.3%)	235 (94.3%)	<0.001
Symptomatic ICH (PH1/PHI2), No. (%)	11 (10.8%)	21 (8.4%)	0.487

NHPI, Native Hawaiian and Pacific Islander; NIHSS, National Institutes of Health Stroke Scale; ASPECTS, Alberta Stroke Program Early CT Score; ICH, intracerebral hemorrhage; PH; parenchymal hemorrhage; mRS, modified Rankin Scale.

In the post-bypass period, the median time from symptom onset to arrival at the CSC (onset-to-door) decreased by more than 60 min compared to the pre-bypass era (67 min vs. 149 min) (Table 2). This was accompanied by a reduction in overall onset-to-reperfusion times (186 min vs. 235 min), despite longer door-to-puncture intervals (76 min vs. 46 min). There was also a five-fold decrease in the proportion of patients transferred from outside hospitals (55.9%vs. 11.6%). Rates of IV thrombolytic therapy did not differ significantly between the two periods.

**Table 2:** Time metric data of pre-LVO bypass vs. Post-LVO bypass thrombectomy patients.

Characteristics	Pre-bypass, *n* = 102	Post-bypass, *n* = 249	*p*-value
Pick up location	*n* = 100	*n* = 247	0.209
Rural, No. (%)	58 (58.0%)	161 (65.2%)	
Urban, No. (%)	42 (42%)	86 (34.8%)	
C-STAT Positive, No. (%)		203 (81.5%)	
Outside Hospital Transfer, No. (%)	57 (55.9%)	29 (11.6%)	<0.001
For OSH transfers:	*n* = 57	*n* = 29	
Onset-to-OSH, min, median (IQR)	59 (42–124)	72 (42–143)	0.505
OSH-to-CSC, min, median (IQR)	109 (89–149)	122 (102–153)	0.075
For all patients:			
Onset-to-CSC Door (OTD), min, median (IQR)	149 (68–222)	67 (46–136)	<0.001
Door-to-Puncture (DTP), min, median (IQR)	46 (26–84)	76 (58–99)	<0.001
Procedure Time, min, median (IQR)	29 (18–40)	21 (14–31)	<0.001
Onset-to-Reperfusion, min, median (IQR)	235 (167–310)	186 (149–250)	0.001
IV thrombolytic treatment, No. (%)	73 (71.6%)	157 (63.1%)	0.127
Onset-to-Needle, min, median (IQR)	102 (71–172)	86 (66–120)	0.039
Door-to-Needle, min, median (IQR)	29 (17–40)	20 (15–32)	0.002
at OSH	37 (29–54), *n* = 38	39 (30–54), *n* = 15	
at CSC	17 (14–30), *n* = 35	19 (14–28), *n* = 142	

OSH, outside hospital; CSC, comprehensive stroke center.

**Figure 1:**
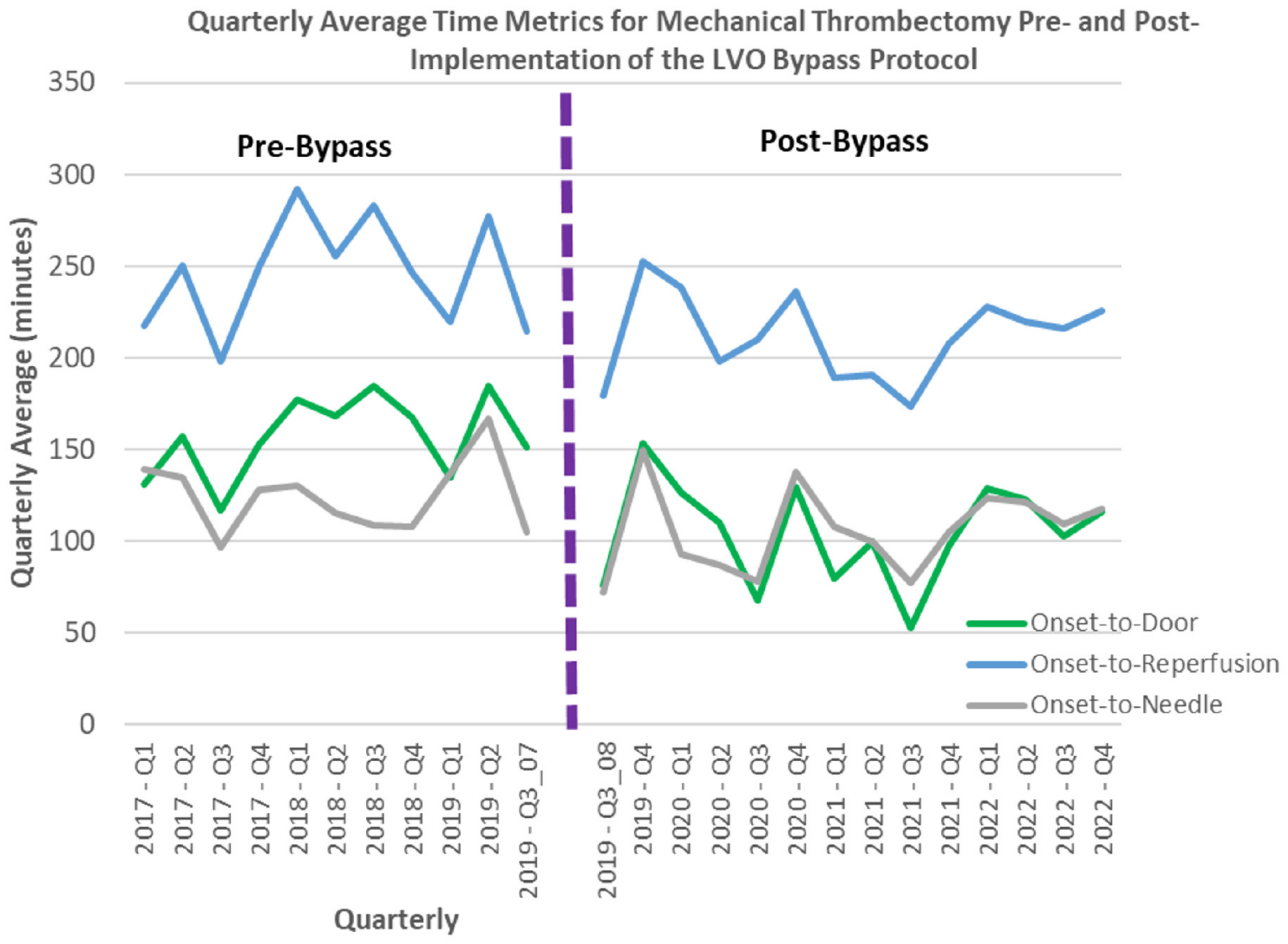
Quarterly average time metrics for mechanical thrombectomy before and after implementation of the LVO bypass protocol.

To account for temporal trends, an interrupted time series (ITS) analysis was performed. ITS modeling demonstrated a significant sustained reduction in the level of onset-to-door times following implementation of the bypass protocol (*p* = 0.0046), corresponding to a 66-min decrease, with no significant change in the pre- or post-intervention slopes (*p* = 0.29 and *p* = 0.38, respectively) (Figure 1; Table 3). Similar level reductions were observed after adjustment for patient-level covariates and maintained over a 36-month post-implementation period. Further ITS modeling also showed a trend toward faster onset-to-reperfusion times in the post-bypass era with a 41-min reduction (*p* = 0.08), without significant delays in onset-to-thrombolytic treatment time (*p* = 0.11) or rates of thrombolytic therapy (*p* = 0.13).

**Table 3:** ITS analysis of time metrics before and after implementation of the LVO bypass protocol.

Onset-to-door (min)	Parameter estimate (SE)	*p*-value
Baseline (Q1 2017)	140.29 (16.88)	
Pre-implementation trend	2.75 (2.49)	0.29
Level change after implementation	−65.97 (20.83)	0.0046
Post-implementation trend	−2.74 (3.03)	0.38
Onset-to-Reperfusion (min)		
Baseline (Q1 2017)	237.75 (18.02)	
Pre-implementation trend	1.33 (2.66)	0.63
Level change after implementation	−41.01 (22.24)	0.080
Post-implementation trend	−1.26 (3.24)	0.71
Onset-to-TPA/TNK (min)		
Baseline (Q1 2017)	123.63 (14.55)	
Pre-implementation trend	0.15 (2.14)	0.95
Level change after implementation	−29.61 (17.96)	0.12
Post-implementation trend	1.20 (2.61)	0.66

In multivariate logistic regression modeling, longer onset-to-reperfusion times were independently associated with worse clinical outcomes, with every 30-min delay increasing the odds of a shift toward a higher 90-day mRS score by approximately 6% (OR 1.061, 95% CI 1.001–1.125, *p* = 0.033; Supplementary Figure 3). The bypass era itself, however, was not independently associated with functional outcomes. Specifically, there was no significant difference in 90-day good outcomes in the pre-bypass vs. the post-bypass eras (mRS score 0–2: 40.2% vs. 44.6%; adjusted OR 0.98 [0.57–1.69]; *p* = 0.93) or a shift in 90-day mRS disability (adjusted OR 0.93 [0.60–1.41]; *p* = 0.72); though a nonsignificant trend toward improved 90-day excellent outcomes was observed (mRS 0–1: 23.5% vs. 34.5%; unadjusted OR 1.75 [1.01–2.90], *p* = 0.045; adjusted OR 1.51 [0.84–2.72], *p* = 0.17; Table 4, Supplementary Figure 4).

**Table 4:** Multivariate logistic regression models for patient outcomes between the pre-LVO bypass and post-LVO bypass periods.

Outcome	Pre-bypass, *n* = 102	Post-bypass, *n* = 249	Unadjusted OR (95%, CI)	*p*-value	Adjusted OR (95% CI)	*p*-value
90-day mRS, median (IQR)	3 (2–4)	3 (1–4)	0.75 (0.50–1.13)	0.172	0.93 (0.60–1.41)^*^	0.722
Good outcome mRS 0-2 at 90d, No. (%)	41 (40.2%)	111 (44.6%)	1.19 (0.75–1.91)	0.452	0.98 (0.57–1.69)^**^	0.932
Excellent outcome mRS 0-1 at 90d, No. (%)	24 (23.5%)	86 (34.5%)	1.75 (1.01–2.90)	0.045	1.51 (0.84–2.72)^**^	0.171

^*^Odds of a higher 90-day mRS in the post-bypass era compared to the pre-bypass era. Ordinal logistic regression model adjusted for age, NIHSS, clot location, reperfusion status, and symptomatic hemorrhage.

^**^Odds of a good or excellent 90-day outcome in the post-bypass era compared to the pre-bypass era. Binary logistic regression model adjusted for age, NIHSS, clot location, reperfusion status, and symptomatic hemorrhage.

In a secondary analysis stratified by pickup location, reductions in treatment times were numerically greater in the post-bypass era among patients presenting from rural locations. Onset-to-door and onset-to-reperfusion times decreased by 95 and 51 min, respectively, among the rural cohort, compared to 39 and 37 min in the urban cohort (Supplementary Figures 8a, 8b). Rates of good and excellent outcomes remained numerically higher in the post-bypass era in both groups, but were not statistically significant.

## Discussion

In this real-world assessment of policy impact, our study demonstrates that the implementation of an island-wide prehospital LVO bypass protocol significantly reduced both onset-to-CSC arrival (onset-to-door) and overall onset-to-reperfusion times among patients treated with mechanical thrombectomy over a 6-year period. In the post-bypass era, patients arrived at the CSC 1 h earlier and achieved successful reperfusion 40 min faster than in the pre-implementation period. This treatment effect was sustained over a 36-month period and largely driven by a 5-fold reduction in the proportion of interfacility transfers.

Despite improvements in treatment times, 90-day good outcomes remained unchanged, consistent with findings from the RACECAT and TRIAGE-Stroke trials. A trend toward improved excellent outcomes was noted, but this association was not significant after adjustment for other predictors of outcome, including higher rates of successful reperfusion and increased medium-vessel occlusions in the post-bypass period.

Several factors may explain the absence of a clinical benefit. First, thrombectomy practices have evolved at our institution, including treatment of patients with higher baseline disability, which may influence patient outcomes over time. Second, although not statistically significant, both “good” (40.2% vs. 44.6%) and “excellent” (23.5 vs. 34.5%) outcomes had numerical improvements in the post-bypass era, suggesting that our study may have been underpowered to detect true differences in functional outcome. As a reference, in TRIAGE-Stroke, the authors estimated that 600 patients were needed to detect a 12% difference in clinical outcomes, which the trial was unable to achieve due to early termination. Our observed 40-min reduction in onset-to-reperfusion times may also have been insufficient to produce a measurable improvement in 90-day outcomes.

Other determinants of patient outcomes such as reperfusion injury (beyond symptomatic hemorrhage), final infarct location, and post-stroke rehab and support, were not captured in our data set, and may have impacted the observed outcomes independent of treatment times and reperfusion success. The time saving benefits of earlier CSC arrival in the post-bypass era were also partially offset by longer door-to-puncture times related to in-house evaluations that previously occurred at the referring hospitals prior to transfer.

Importantly, because our bypass protocol was implemented across the entirety of the island's EMS system, we included all patients evaluated by EMS regardless of location, and did not restrict to only those who were true bypasses (i.e., patients located further from the CSC and who truly bypassed a local facility). As a study on real-world impact, this was intended to avoid selection bias, but may have diluted the clinical effect by including patients who would have been transported directly to the CSC regardless.

Although the bypass era itself was not independently associated with patient outcomes, delays in “onset-to-reperfusion” time remained a predictor of worse 90-day outcomes, reiterating the importance of timely care. Specifically, every 30-min delay to reperfusion increased the odds of a higher 90-day mRS score by 6%. Since the implementation of the bypass protocol, our data shows that there continues to be opportunities to improve prehospital systems of care, where onset-to-door times still exceed 60 min. Efforts to shift stroke care into the prehospital phase are already underway, including implementation of mobile stroke units to deliver IV thrombolytics (Grotta et al., 2021); use of head impulse monitors to detect LVOs in the field (Paxton et al., 2024); and the development of telemedicine-enabled ambulances to provide real-time access to neurology specialists (Harahsheh et al., 2024).

Prior studies have raised concerns about EMS bypass protocols delaying access to thrombolytic therapy (Schlemm et al., 2020). Within our stroke system, there were no significant differences in the rates of intravenous thrombolytic administration or in onset-to-thrombolytic treatment times when patients bypassed local hospitals. This may be attributable to faster door-to-needle times at the receiving CSC, reflecting its high-volume status and continuous in-person stroke coverage, factors that offset delays related to longer prehospital transport.

Interestingly, there was a higher proportion of favorable pre-treatment ASPECTS in the post-bypass era. This contrasts with national trends, including at our own institution, where treatment of larger core infarcts has become increasingly common following recent large core thrombectomy studies (Sarraj et al., 2023; Li et al., 2023). Factors that may influence this finding include: 1) The study period concluded on January 1, 2023, predating our institutions transition toward treating large core infarcts; and 2) patients in the extended 8–24-h time window who are more likely to have large core infarcts were excluded, under the assumption that delays to presentation (i.e., wake-up strokes or late symptom recognition) are not modifiable by EMS transport policies. While beyond the scope of the study, it is possible that reductions in onset-to-door times in the post-bypass era contributed to improved pre-treatment ASPECTS by reducing delays prior to imaging.

Our study had several limitations. First, our results are not generalizable to other stroke networks given that the entire island's thrombectomy-capabilities are serviced by a single CSC within a closed geographic region. Second, a greater number of patients were treated in the post-bypass era, reflecting institutional changes in stroke recognition and patient selection. Beginning in 2017, there were hospital initiatives that lowered the threshold for stroke code activations, and wider adoption of RAPID AI software to streamline imaging acquisition and decision making. Concurrent with these changes, institutional stroke code activations consistently increased over time (Supplementary Figure 5). Patient selection also shifted, where the number of proximal M2 occlusions treated increased from 4 in 2018 to 25 in 2022 (Supplementary Figure 6). These temporal changes may have influenced treatment workflows and thrombectomy volumes, independent of the bypass protocol.

Third, the study only looked at patients treated with mechanical thrombectomy, and thus, does not address how the LVO bypass protocol influenced patient eligibility for treatment, nor the rates of false positive “stroke mimics.” In a *post hoc* analysis of a 5-month period between 2019 to 2020, 54% of C-STAT positive patients did not have an LVO, which were comprised of intracerebral hemorrhages, small vessel strokes, or other stroke mimics (Supplementary Figures 7a, 7b). This highlights the potential unintended consequence of bypass protocols overburdening the ED and its resources. Within our regional system of care, however, a non-LVO transport does not necessarily represent an unnecessary transport, as patients with intracerebral hemorrhages or status epilepticus may ultimately require transfer to our Neuroscience ICU for specialized care.

Although the study encompassed the COVID-19 pandemic, a sensitivity analysis excluding the major winter 2020–2021 surge demonstrated that reductions in onset-to-door and onset-to-reperfusion times remained statistically significant in the post-bypass era with comparable treatment times to the primary analysis. This suggests that pandemic related disruptions to EMS and ED workflow did not alter the overall observations from the primary study.

In this pre-implementation vs. post-implementation study of an island-wide EMS LVO bypass protocol, we observed a sustained and measurable reduction in both onset-to-door and onset-to-reperfusion treatment times among patients treated with mechanical thrombectomy for stroke. While we did not observe statistically significant improvements in patient outcomes in the post-bypass era despite net reductions in CSC arrival time, the overall time from onset to reperfusion remains a clinical predictor of disability. It remains to be seen whether other opportunities to improve treatment times, beyond EMS bypass protocols, may yield clinical outcome benefit.

## Data Availability

The raw data supporting the conclusions of this article will be made available by the authors, without undue reservation.

## References

[B1] Almekhlafi M. A. Goyal M. Dippel D. W. J. Majoie C. B. L. M. Campbell B. C. V. Muir K. W. . (2021). Healthy life-year costs of treatment speed from arrival to endovascular thrombectomy in patients with ischemic stroke: a meta-analysis of individual patient data from 7 randomized clinical trials. JAMA Neurol. 78, 709–717. doi: 10.1001/jamaneurol.2021.1055 33938914 PMC8094030

[B2] Behrndtz A. Blauenfeldt R. A. Johnsen S. P. Valentin J. B. Gude M. F. Al-Jazi M. A. . (2023). Transport strategy in patients with suspected acute large vessel occlusion stroke: TRIAGE-STROKE a randomized clinical trial. Stroke 54, 2714–2723. doi: 10.1161/STROKEAHA.123.043875 37800374 PMC10589426

[B3] Froehler M. T. Saver J. L. Zaidat O. O. Jahan R. Aziz-Sultan M. A. Klucznik R. P. . (2017). Interhospital transfer before thrombectomy is associated with delayed treatment and worse outcome in the stratis registry. Circulation. 136, 2311–2321. doi: 10.1161/CIRCULATIONAHA.117.028920 28943516 PMC5732640

[B4] Grotta J. C. Yamal J. M. Parker S. A. Rajan S. S. Gonzales N. R. Jones W. J. . (2021). Prospective, multicenter, controlled trial of mobile stroke units. N Engl J Med 385, 971–981. doi: 10.1056/NEJMoa2103879 34496173

[B5] Harahsheh E. English S. W. Jr. Demaerschalk B. M. (2024). Telemedicine-enabled ambulances for prehospital acute stroke management: a pilot study. Mayo Clin Proc Digit Health. 2, 533–541. doi: 10.1016/j.mcpdig.2024.08.006 40206528 PMC11975842

[B6] Jahan R. Saver J. L. Schwamm L. H. Fonarow G. C. Liang L. Matsouaka R. A. . (2019). Association between time to treatment with endovascular reperfusion therapy and outcomes in patients with acute ischemic stroke treated in clinical practice. JAMA 322, 252–263. doi: 10.1001/jama.2019.8286 31310296 PMC6635908

[B7] Jayaraman M. V. Hemendinger M. L. Baird G. L. Yaghi S. Cutting S. Saad A. . (2020). Field triage for endovascular stroke therapy: a population-based comparison. J Neurointerv Surg. 12, 233–239. doi: 10.1136/neurintsurg-2019-015033 31484698

[B8] Kass-Hout T. Lee J. Tataris K. Richards C. T. Markul E. Weber J. . (2021). Prehospital comprehensive stroke center vs. primary stroke center triage in patients with suspected large vessel occlusion stroke. JAMA Neurol. 78, 1220–1227. doi: 10.1001/jamaneurol.2021.2485 34369969 PMC8353578

[B9] Li Q. Abdalkader M. Siegler J. E. Yaghi S. Sarraj A. Campbell B. C. V. . (2023). Mechanical thrombectomy for large ischemic stroke: a systematic review and meta-analysis. Neurology 101, e922–e932. doi: 10.1212/WNL.0000000000207536 37277200 PMC10501098

[B10] Mazighi M. Chaudhry S. A. Ribo M. Khatri P. Skoloudik D. Mokin M. . (2013). Impact of onset-to-reperfusion time on stroke mortality: a collaborative pooled analysis. Circulation 127, 1980–1985. doi: 10.1161/CIRCULATIONAHA.112.000311 23671178

[B11] Paxton J. H. Keenan K. J. Wilburn J. M. Wise S. L. Klausner H. A. Ball M. T. . (2024). Headpulse measurement can reliably identify large-vessel occlusion stroke in prehospital suspected stroke patients. Acad Emerg Med 31, 848–859. doi: 10.1111/acem.14919 38643419

[B12] Pérez de la Ossa, N. Abilleira S. Jovin T. G. (2022). Effect of direct transportation to thrombectomy-capable center vs. local stroke center on neurological outcomes in patients with suspected large-vessel occlusion stroke in nonurban areas: the RACECAT Randomized Clinical Trial. JAMA 327, 1782–1794. doi: 10.1001/jama.2022.4404 35510397 PMC9073661

[B13] Sarraj A. Hassan A. E. Abraham M. G. Ortega-Gutierrez S. Kasner S. E. Hussain M. S. . (2023). Trial of endovascular thrombectomy for large ischemic strokes. N Engl J Med 388, 1259–1271. doi: 10.1056/NEJMoa2214403 36762865

[B14] Saver J. L. Goyal M. van der Lugt A. Menon B. K. Majoie C. B. Dippel D. W. . (2016). Time to treatment with endovascular thrombectomy and outcomes from ischemic stroke: a meta-analysis. JAMA 316, 1279–1288. doi: 10.1001/jama.2016.13647 27673305

[B15] Schlemm L. Endres M. Nolte C. H. (2020). Bypassing the closest stroke center for thrombectomy candidates: what additional delay to thrombolysis is acceptable? Stroke 51, 867–875. doi: 10.1161/STROKEAHA.119.027512 31964288

[B16] Sun C. H. Nogueira R. G. Glenn B. A. Connelly K. Zimmermann S. Anda K. . (2013). “Picture to puncture”: a novel time metric to enhance outcomes in patients transferred for endovascular reperfusion in acute ischemic stroke. Circulation 127, 1139–1148. doi: 10.1161/CIRCULATIONAHA.112.000506 23393011

[B17] Sun C. H. Zaidat O. O. Castonguay A. C. Veznedaroglu E. Budzik R. F. English J. . (2023). A decade of improvement in door-to-puncture times for mechanical thrombectomy but ongoing stagnation in prehospital care. Stroke Vas. Intervent. Neurol. 3:e000561. doi: 10.1161/SVIN.122.000561 41585762 PMC12778601

[B18] Wagner A. K. Soumerai S. B. Zhang F. Ross-Degnan D. (2002). Segmented regression analysis of interrupted time series studies in medication use research. J Clin Pharm Ther. 27, 299–309. doi: 10.1046/j.1365-2710.2002.00430.x 12174032

