# Supplementary material for: Reducing onset-to-door times through the implementation of a prehospital large vessel occlusion bypass protocol: the Oahu experience
Source: Front Stroke. 2026 Sep 3;5:1903852. doi: 10.3389/fstro.2026.1903852 (PMC13581692; doi:10.3389/fstro.2026.1903852)
Supplement: Supplementary file 1 [file Table_1.docx]

**Supplemental 1: Regional Stroke Network on Oahu**

Map of Oahu showing the location of ten regional hospitals and one thrombectomy capable Comprehensive Stroke Center.

**Supplemental 2: Patient Selection for Study**

**Supplemental 3: Ordinal Logistic Regression Model for Predictors of Higher 90-day modified Rankin Scale score**

| **Predictor** | **Adjusted OR (95% CI)** | **P-value** |
| --- | --- | --- |
| Baseline NIHSS (per point) | 1.077 (1.047 – 1.108) | <0.001 |
| Age (per year) | 1.037 (1.021 – 1.051) | <0.001 |
| Clot location: *M2 as reference |  |  |
| ICA-T vs M2 | 1.93 (1.11 – 3.34) | 0.02 |
| M1 vs M2 | 1.15 (0.71 – 1.85) | 0.56 |
| Failed reperfusion | 4.45 (2.21 – 8.95) | <0.001 |
| Absence of symptomatic hemorrhage | 0.29 (0.14 – 0.56) | <0.001 |
| Onset-to-Reperfusion (per 30 mins) | 1.062 (1.001 – 1.13) | 0.033 |

Abbreviation: ICA-T, internal carotid artery terminus; M1, middle cerebral artery M1 segment; M2, middle cerebral artery M2 segment.

**Supplemental 4:** Distribution of the modified Rankin Scale (mRS) scores at 90-day for all patients treated with mechanical thrombectomy

P-values reflect the odds of a higher 90-day mRS in the post-bypass era compared to the pre-bypass era. The adjusted model accounted for age, NIHSS, clot location, reperfusion status, and symptomatic hemorrhage using ordinal logistic regression.

**Supplemental 5:**

**Supplemental 6:**

**Supplemental 7a:**

**Supplemental 7b:**

**Supplemental 8a:**

| **Time Metric Data of Pre-LVO Bypass vs. Post-LVO Bypass Thrombectomy Patients;**  **Rural Pick-Up Location Only (n=219)** | | | |
| --- | --- | --- | --- |
|  |  |  |  |
|  | Pre-Bypass, n=58 | Post-Bypass, n=161 | P-value |
|  |  |  |  |
| **C-STAT Positive, No. (%)** |  | 135 (83.4%) |  |
|  |  |  |  |
| **Outside Hospital Transfer, No. (%)** | 47 (81.0%) | 24 (14.9%) | <0.001 |
|  |  |  |  |
| **Onset-to-CSC Door (OTD), median (IQR)** | 169 (130-239) | 74 (51-161) | <0.001 |
| **Door-to-Puncture (DTP), median (IQR)** | 30 (21-64) | 74 (57-97) | <0.001 |
| **Procedure Time, mins, median (IQR)** | 31 (20-41) | 21 (14-32) | <0.001 |
| **Onset-to-Reperfusion, median (IQR)** | 248 (195-322) | 197 (155-261) | <0.001 |
|  |  |  |  |
| **IV Thrombolytic Treatment, No. (%)** | 42 (72.4%) | 101 (62.7%) | 0.184 |
| **Onset-to-Needle, mins, median (IQR)** | 117 (81-185) | 90 (68-143) | 0.050 |
| **Door-to-Needle, mins, median (IQR)** | 36 (26-50) | 22 (16-32) | <0.001 |
|  |  |  |  |
| **Good outcome mRS 0-2 at 90d, No. (%)** | 22 (37.9%) | 72 (44.7%) | 0.370 |
| **Excellent outcome mRS 0-1 at 90d, No. (%)** | 15 (25.8%) | 56 (34.8%) | 0.213 |

Abbreviation: OSH, outside hospital; CSC, Comprehensive Stroke Center

**Supplemental 8b:**

| **Time Metric Data of Pre-LVO Bypass vs. Post-LVO Bypass Thrombectomy Patients;**  **Urban Pick-Up Location Only (n=128)** | | | |
| --- | --- | --- | --- |
|  |  |  |  |
|  | Pre-Bypass, n=42 | Post-Bypass, n=86 | P-value |
|  |  |  |  |
| **C-STAT Positive, No. (%)** |  | 67 (78.0%) |  |
|  |  |  |  |
| **Outside Hospital Transfer, No. (%)** | 10 (23.8%) | 4 (4.6%) | <0.001 |
|  |  |  |  |
| **Onset-to-CSC Door (OTD), median (IQR)** | 92 (40-185) | 53 (39-90) | 0.095 |
| **Door-to-Puncture (DTP), median (IQR)** | 70 (45-94) | 81 (63-101) | 0.034 |
| **Procedure Time, mins, median (IQR)** | 27 (17-32) | 14 (14-31) | 0.154 |
| **Onset-to-Reperfusion, median (IQR)** | 205 (152-303) | 168 (143-224) | 0.189 |
|  |  |  |  |
| **IV Thrombolytic Treatment, No. (%)** | 30 (71.4%) | 55 (64.0%) | 0.401 |
| **Onset-to-Needle, mins, median (IQR)** | 82 (61-171) | 74 (59-101) | 0.193 |
| **Door-to-Needle, mins, median (IQR)** | 23 (14-34) | 17 (14-29) | 0.445 |
|  |  |  |  |
| **Good outcome mRS 0-2 at 90d, No. (%)** | 17 (40.5%) | 38 (44.2%) | 0.691 |
| **Excellent outcome mRS 0-1 at 90d, No. (%)** | 8 (19.0%) | 29 (33.7%) | 0.086 |

Abbreviation: OSH, outside hospital; CSC, Comprehensive Stroke Center
